# Assessment of psychometric properties of the self-stigma inventory for Iranian families of persons who use drugs

**DOI:** 10.3389/fpubh.2022.1017273

**Published:** 2022-11-07

**Authors:** Mohammadreza Dinmohammadi, Amir Jalali, Arsalan Naderipour

**Affiliations:** ^1^Department of Nursing, School of Nursing and Midwifery, Zanjan University of Medical Sciences, Zanjan, Iran; ^2^Substance Abuse Prevention Research Center, Research Institute for Health, Kermanshah University of Medical Sciences, Kermanshah, Iran; ^3^Department of Emergency Medicine, Faculty of Paramedics, Kermanshah University of Medical Sciences, Kermanshah, Iran

**Keywords:** reliability, validity, stigma, drug user, family, substance use disorder

## Abstract

**Background:**

Substance use disorder (SUD) and its related problems take a toll on the individual, family, and society. This study was conducted to determine the psychometric properties of the self-stigma scale in the families of persons who use drugs (PWUDs) in Iran.

**Methods:**

This was a methodological and psychometric study. The study population consisted of 311 family members of PWUDs visiting outpatient and inpatient addiction treatment centers in Kermanshah who were selected using convenience sampling. The 14-item Self-Stigma Inventory for Families (SSI-F), which was developed by Yildiz et al. in 2019 using interviews and scales connected with stigma, was applied. The ten steps developed by Wilde et al. were used in this study for cultural validation. The exploratory factor analysis (EFA) (140 samples) and confirmatory factor analysis (CFA) (311 subjects) were used to confirm the construct validity, and the test-retest method was used to confirm the reliability of the tools. Cronbach's alpha was also used to test the internal consistency of the tools.

**Findings:**

The results of EFA and CFA scales in families of PWUDs were confirmed with three factors and 14 items. The reliability degree of the tools was confirmed as 0.891 and the Cronbach's alpha was confirmed as 0.879 using the test-retest method. Pearson's correlation coefficient indicated a positive and significant status between the scale's items/factors and the scale itself.

**Conclusion:**

Generally, the results showed that the PWUDs SSI-F scale in Iranian families was valid and reliable with three factors and 14 items, and it can be used to conduct relevant studies.

## Introduction

Stigma is a set of negative beliefs held by a particular group or society about a particular subject or people (1). Stigma is rarely based on facts, but it is mainly based on assumptions and generalities (2). It can lead to prejudice, avoidance, rejection, and discrimination against people having undesirable social qualities or marginal cultural behaviors like substance abuse (3). Self-stigma is a gradual process where a person assumes the same negative attitude toward some qualities without criticizing the negative social prejudices against those qualities (4). The results of the study indicate a public negative attitude toward people taking drugs which is even more negative than the attitude toward people suffering from schizophrenia (5).

The stigma of taking drugs is one of the greatest obstacles for people who seek treatment and are being treated for substance abuse disorders (6). The variables of mental health (the temptation to use drugs, depression, anxiety, and life quality) in connection with persons who use drugs (PWUDs) have the strongest relationship with self-stigma (7). Although there are a number of stigmas attached to PWUDs, all people dealing with substance abuse do not experience the same number of stigmas (8). The results of the study conducted by Stringer showed that married parents suffer from the highest level of self-stigma, and the family member of PWUDs suffer from the greatest mental pressure exerted by stigma (9). Stigma is attached to families of PWUDs through interaction with neighbors, extended family, and also healthcare personnel. The inappropriate attitude of relatives, neighbors, and other people in society toward PWUDs is the main factor involved in the attachment of stigma to the families and PWUDs (10).

Generally, healthcare experts hold a negative attitude toward PWUDs (11). They consider violence, manipulation, and low motivation as the factors preventing the provision of treatment services to these patients (9, 11). Attaching stigma to PWUDs is a prevalent phenomenon that has destructive effects on the treatment results, healthcare staff, treatments, research, policies, and society in general (2). The negative attitude of healthcare experts decreases the power to rehabilitate patients and weakens treatment results (11). The results of the studies have indicated the willingness of the families and PWUDs to form supportive relationships with other people and to cooperate in treatment and caretaking (12). Therefore, it is necessary to plan and implement appropriate actions to improve the interaction of help-seekers' families with other people, society, and healthcare personnel by measuring the degree of perceived stigma and self-stigma in these families (13). Concerning the measurement of the degree of stigma in people suffering from mental illnesses and their families (14–16), PWUDs (17) were introduced. However, a standard tool, which is both valid and reliable, to evaluate self-stigma in families of PWUDs in Iran should be prepared and used for education, as well as healthcare and treatment programs. Families of the patients with schizophrenia (Self-Stigma Inventory for Families [SSI-F]) scale has been developed in 2019 by Yildiz et al. with the same purpose, which includes 14 questions and 3 factors (social withdrawal, concealment, and low validity) (16). Considering that substance use disorders (SUDs) are subcategories of mental and behavioral disorders, and also by examining the items of the abovementioned tool, it is clear that due to Turkey's proximity to Iran, the items are very close to the norms and culture in Iran. Therefore, this questionnaire can be an appropriate tool to evaluate self-stigma in families of PWUDs. This study was conducted to determine the psychometrics of the stigma questionnaire in families of PWUDs in Iran.

## Materials and methods

### Design and setting

This was a methodological and validation study. The study population consisted of the family members of PWUDs visiting the addiction treatment centers in Kermanshah. In total, 22 private and public substance abuse treatment centers in Kermanshah city were selected. The research units were also selected from the family members of patients undergoing maintenance treatment with methadone in the clinics who had a history of using natural drugs such as opium, heroin, and crack.

### Participants

In total, 311 (18) subjects were selected using convenience sampling and according to the inclusion criteria among the blood relatives (children, parents, and peers) of PWUDs who visited the private or public addiction treatment centers in Kermanshah.

### Participant's selection method

The sample size in the validity stage of the construct [exploratory factor analysis (EFA) = 140] in confirmatory factor analysis (CFA) and the reliability of 311 subjects were used (18, 19). Being an immediate family member of PWUD, having an interest in participation, and having an age of 18–54 years were considered the inclusion criteria while completing < 90% of the questionnaire was considered an exclusion criterion in this study.

### Research instrument

The demographic form of the patients and their family members who participated in the study and the SSI-F were the main tools used in the study.

#### Self-stigma inventory for families

The 14-item SSI-F developed by interviews and scales connected with stigma in 2019 evaluates self-stigma in the families of people having mental illnesses. This scale was designed using focus group interviews and the study of existing scales. Initially, it had 19 items that were validated in Turkish society and then reduced to 14 items. The scale was designed based on a Likert scale, and each item included five options, namely, (1) not agree, (2) slightly agree, (3) somewhat agree, (4) generally agree, and (5) completely agree. The SSI-F has a Cronbach's alpha that equals 0.88 and a reliability coefficient that equals 0.93 based on the test-retest method in Turkish society (16).

### Cultural validation

After obtaining permission from the designer of the tools, the ten steps proposed by Wild were used to translate and validate the tools culturally (20).

- Step 1: Key native people (proficiency in English-Farsi, Iran residency, and previous experience of translating texts into the mentioned languages) were selected to render translations.
- Step 2: Separate translation of the SSI-F scale to Farsi by two individuals.
- Step 3: Holding a panel consisting of the research team and a combination of two initial Farsi translations into one single translation. At this stage, the schizophrenia disorder in the subjects was changed to SUD.
- Step 4: Returning the final version translated from Farsi into the original language of the tools by two translators independent of the second step translators.
- Step 5: Two translations provided by the fourth step were examined by the research team to make sure of the conceptual equality of the translations.
- Step 6: The research team made the conceptual comparison of the versions produced in the fifth step with the original scale. Finally, a single version was prepared in the original language, the tools were sent to the designer of the tools to obtain his views, and his views were implemented.
- Step 7: A final version (in Farsi) was provided to 16 family members of PWUDs to examine cognitive equality, and their abilities to understand, interpret, and perceive were evaluated.
- Step 8: The tools were reviewed according to the results obtained from the cognitive information to make sure of cultural adaptation.
- Step 9: Farsi version of the tools was controlled for any typos or grammatical errors.
- Step 10: Work process and the reported final version.

### Data analysis

The face validity was examined using the views of 16 family members of PWUDs, and the quantitative and qualitative content validity was examined using the views of 16 researchers and experts (four psychiatric nursing and clinical psychology faculty members and four public health faculty members). Then, the quantitative content validity (21) of the tools was calculated for each item according to Walts & Basel index method (Table 1). The test-retest tool was used to examine the reliability of the tools (22), and Cronbach's alpha was used to test the internal consistency of the tools. All statistical analyses were carried out using SPSS 25 and LISREL 8.

**Table 1:** The ratio and index of content validity, and multivariate normality index of the tool items.

		**Skewness^d^**	**Kurtosis^c^**	**CVI^b^**	**CVR^a^**
1	I think people are worried that I may lose my control since I am a family member of a PWUD.	0.19	−1.23	0.92	0.83
2	I try to avoid individuals who may hurt me by their opinions and words since I am a family member of a PWUD.	0.16	−1.31	0.92	0.66
3	I think people do not care about me, because I am a family member of a PWUD.	−0.02	−1.23	0.83	0.66
4	I think I am a burden on others, because I am a family member of a PWUD.	0.18	−1.24	0.83	0.83
5	I think I cannot make right decisions, because I am a family member of a PWUD.	0.18	−1.25	0.92	0.83
6	Since I think that others do not understand me, I tend to avoid them, because I am a family member of a PWUD.	0.09	−1.28	0.92	0.66
7	I do not tell others what the actual name of my kin's disease is, because I am afraid that they might desert me.	0.19	−1.25	0.92	0.83
8	I do not tell my relatives what the actual name of my kin's SUD is.	0.14	−1.33	0.92	0.83
9	I do not tell my friends that one of my family members has a SUD	0.01	−1.34	0.83	0.83
10	I feel less self–esteem since I have started to live with a PWUD	0.15	−1.32	0.83	1
11	I feel useless as I am part of a family with a PWUD.	0.19	−1.26	0.92	0.66
12	I think I cannot be a successful person, because I am a family member of a PWUD.	0.23	−1.26	1	0.66
13	I think I cannot be happy, because I am a family member of a PWUD.	0.28	−1.19	0.92	1
14	I cannot be as responsible as others, because I am a family member of a PWUD.	0.20	−1.28	1	0.66
	**SSI–F (PWUDs)**			0.91	0.78
		**Mardia test** **=** **126.36**			

a Content Validity Ratio,

b Content Validity Index,

c Skewness is a measure of symmetry, or more precisely, the lack of symmetry,

d Kurtosis is a measure of whether the data are heavy–tailed or light–tailed relative to a normal distribution.

## Results

### Descriptive results

The average age of PWUDs was 66.37 (±11.59) years with a minimum age of 18 years and a maximum age of 63 years, and the average age of the family members was 36.61 (11.25) years with a minimum age of 18 years and a maximum age of 63 years (Table 2).

**Table 2:** Demographic characteristics of the study participants.

**Variable**		**EFA (140)** ***N* (%)**	**CFA (311)** ***N* (%)**
Gender (PWUDs)	Male	133(95)	299(96.1)
	Female	7(5)	12(3.9)
Gender (Family member)	Male	84(60)	137(38.6)
	Female	56(40)	218(61.4)
Marital status (Family member)	Married	78(55.7)	159(51.1)
	Single	62(44.3)	152(48.9)
Educational level (Family member)	Elementary level	13(9.3)	36(11.6)
	Secondary level	25(17.9)	57(18.3)
	High school diploma	81(57.9)	154(49.5)
	Higher education	21(15)	64(20.6)
Domicile (Family member)	City	89(63.9)	205(65.9)
	Suburb	48(34.3)	99(31.8)
	Rural area	3(2.1)	7(2.3)
Job (Family member)	Unemployed	29(20.7)	58(18.6)
	Employed	38(27.1)	86(27.7)
	Manual worker	27(19.3)	60(19.3)
	Freelancer	27(19.3)	40(12.9)
	House wife	19(13.6)	67(21.5)
Relation of PWUD	Spouse	14(10)	32(10.3)
	Children	25(17.9)	73(23.5)
	Brother	40(28.6)	91(29.3)
	Sister	35(25)	62(19.9)
	Parents	26(18.6)	53(17)
Drug use duration (PWUDs)	<1 year	16(11.4)	
	1–3 years	24(17.1)	
	3–5 years	25(17.9)	
	More than 5 years	75(53.6)	

### Construct validation results

#### Exploratory factor analysis (EFA)

Exploratory factor analysis was conducted on the 140 initial samples. First, the correlation coefficients of the scores of questionnaire items were examined, and it was assured that they were high. The results of Kaiser–Meyer–Olkin (KMO) and Bartlett's test of sphericity were used for this purpose (KMO = 0.841, chi-square = 751.072, P_value_ = 0.0001). Considering the values of KMO, carrying out EFA on this questionnaire was justifiable.

After making sure of the above assumptions, EFA was carried out on the subjects' answers and 14 items of the questionnaire. In this research, the principal component (PC) and Varimax rotation analysis methods were used to extract the factors. The shared values of each question were extracted using the PC analysis, and the results of their reliability test are shown in Table 3.

**Table 3:** Extracted eigenvalues for each sol and stability test.

	**Extraction communalities**	**Corrected item–total correlation**	**Cronbach's–alpha if item deleted**
Q1	0.586	0.535	0.787
Q2	0.457	0.534	0.787
Q3	0.566	0.635	0.765
Q4	0.423	0.491	0.798
Q5	0.662	0.679	0.756
Q6	0.612	0.553	0.784
Q7	0.535	0.438	0.576
Q8	0.711	0.553	0.413
Q9	0.499	0.387	0.642
Q10	0.540	0.599	0.795
Q11	0.546	0.619	0.789
Q12	0.637	0.658	0.778
Q13	0.711	0.714	0.761
Q14	0.510	0.506	0.820

**Figure 1:**
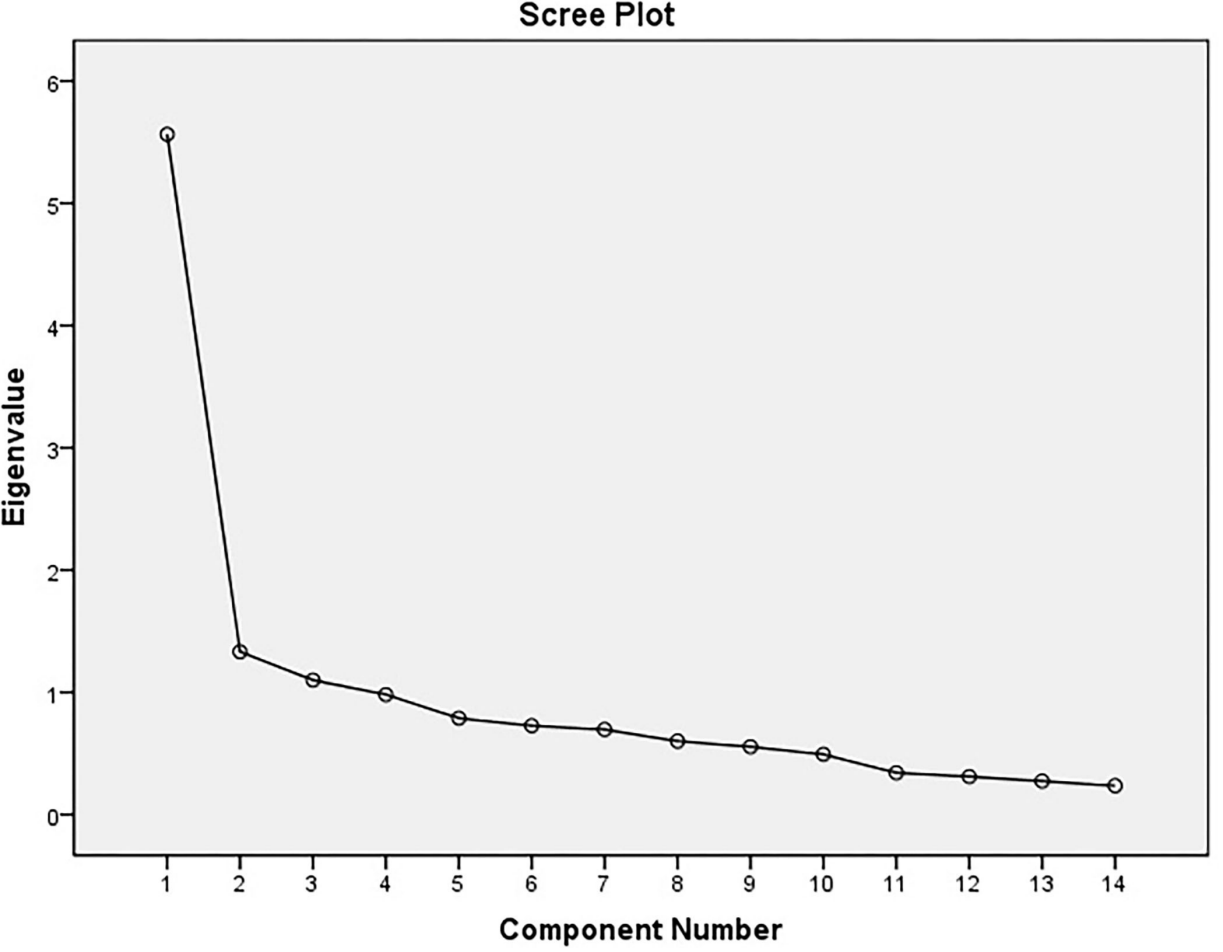
Cattell's scree plot of the extracted elements of the scale.

Then, to determine the number of factors, the factors whose percentage of specific value was > 1 were selected. The initial results showed that 3 factors or components can be selected to be analyzed. In Table 4, the extracted factors are shown along with the special values and the percentage of each factor's share in accounting for the variance of 14 items. The cumulative variance explained by each one of the 3 factors has been presented (Figure 1).

**Table 4:** Factors extracted after exploratory analysis.

**Component**	**Initial eigenvalues**			**Extraction sums of squared loadings**			**Rotation sums of squared loadings**		
	**Total**	**% of Variance**	**Cumulative %**	**Total**	**% of Variance**	**Cumulative %**	**Total**	**% of Variance**	**Cumulative %**
1	5.564	39.745	39.745	5.564	39.745	39.745	3.261	23.294	23.294
2	1.332	9.512	49.257	1.332	9.512	49.257	2.704	19.317	42.612
3	1.100	7.857	57.114	1.100	7.857	57.114	2.030	14.503	57.114
4	0.981	7.010	64.125						
5	0.789	5.633	69.758						
6	0.727	5.192	74.950						
7	0.696	4.969	79.919						
8	0.601	4.294	84.213						
9	0.555	3.963	88.175						
10	0.493	3.520	91.695						
11	0.342	2.443	94.138						
12	0.310	2.218	96.356						
13	0.274	1.957	98.312						
14	0.236	1.688	100.000						

Annex Table 1 shows the rotated factor matrix. In this table, the questions with factor loadings > 0.3 and the greatest loading were loaded on the given component. According to the results presented in Supplementary Table 1, the extracted factors have been presented along with the items of each factor in Table 5.

**Table 5:** *T*–value, factor loadings, correlation, and Cronbach's alpha of the tool items.

**Factor**	**No**	**T^a^**	**λ^b^**	**Correlation coefficient**	**Cronbach's alpha**	
Social withdrawal (SW)	S1	13.05	0.69^***^	0.565^**^	0.787	0.810
	S2	11.11	0.61^***^	0.6^**^	0.787	
	S3	13.47	0.71^***^	0.659^**^	0.765	
	S4	13.09	0.69^***^	0.635^**^	0.798	
	S5	14.86	0.76^***^	0.71^**^	0.756	
	S6	13.11	0.69^***^	0.64^**^	0.784	
Concealment of the illness (CI)	S7	13.74	0.77^***^	0.568^**^	0.576	0.647
	S8	13.22	0.74^***^	0.546^**^	0.413	
	S9	11.71	0.67^***^	0.497^**^	0.642	
Perceived devaluation (PD)	S10	13.25	0.70 ^***^	0.649^**^	0.795	0.824
	S11	13.92	0.73^***^	0.64^**^	0.789	
	S12	13.57	0.71^***^	0.63^**^	0.778	
	S13	15.3	0.78^***^	0.68^**^	0.761	
	S14	12.81	0.68^***^	0.62^**^	0.820	
SSI–F (PWUDs)						0.897

^**^P_value_ < 0.01

*** P_value_ < 0.001.

a The calculated values of t for all factor loadings of the first and second order are > 1.96 and are therefore significant at the 95% confidence level,

b The specific value, which is denoted by the Lambda coefficient and the statistical symbol λ, is calculated from the sum of the factors of the factor loads related to all the variables of that factor.

#### Confirmatory factor analysis (CFA)

*Confirmatory factor analysis* was carried out for 311 samples. Mardia's test using skewness and kurtosis was used to confirm the multivariate normality of data distribution (values ranged from −2 to +2) where the statistic of Mardia's test was 126.36. As to multivariate normality, Mardia's test was used so that multivariate normality is rejected if the critical ratio (CR) for skewness is, < 7 (23, 24). EFA and CFA were used to confirm the construct validity (Table 1).

**Figure 2:**
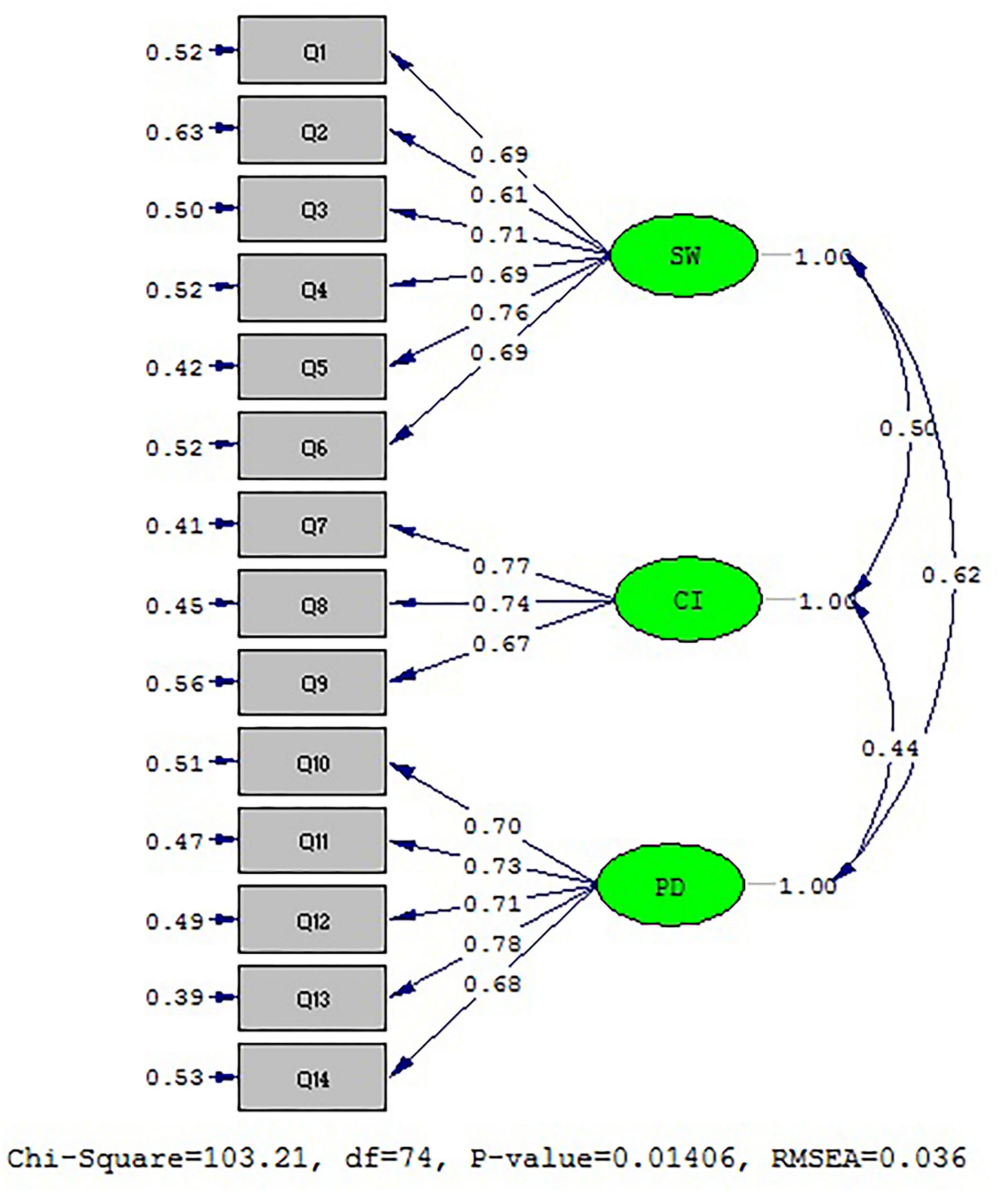
Three factor models of SSI_F in Iranian family members of PWUDs.

The results of the factor analysis test to determine standard coefficients are presented in Figure 2. Regarding the fact that all values of factor loadings and t vibration were greater than the critical value of 1.96, there was no need to remove any item (Table 5). Moreover, indices of CFA model fit are presented in Table 6. In contrast, the model fit is appropriate considering the fit indices shown in the above table. Therefore, the above model fits with the obtained data.

**Table 6:** Fit indicators confirmatory factor analysis SSI–F (PWUDs).

**Fit indicators**	**Criterion**	**Level**	**Interpretation**
χ^2^/DF	3 ≤	1.39	Optimal fit
CFI	0.9<	0.99	Optimal fit
NNFI/TLI	0.9<	0.99	Optimal fit
GFI	0.8 <	0.93	Optimal fit
RMSEA	0.08>	0.036	Optimal fit
R^2^	Near to 1	0.99	Optimal fit
SRMR	0.05>	0.033	Optimal fit
	DF = 74 *p*–value = 0.093; Chi–Square = 103.21		

The reliability of the tools was obtained using the test-retest method and 15 individuals independent of the original sample who had completed the Farsi version (SSI-F of PWUDs) again after 10 days, and the value was obtained as r = 0.891. Cronbach's alpha was calculated to examine the internal reliability (internal validity) of the Farsi version of SSI-F of PWUDs and it was obtained as 0.879 for the total index of 14 items. The validity coefficient was obtained from 0.647 to 0.824 using Cronbach's alpha for the subscales of the Farsi version of SSI-F of PWUDs. Therefore, the subscales enjoy the required reliability to be assessed (Table 6). Moreover, Pearson's test showed that there was a positive and significant relationship between the subscales and the main scale (Table 7).

**Table 7:** Correlation coefficients of SSI–F (PWUDs) factors together.

	**Social withdrawal**	**Concealment of the illness**	**Perceived devaluation**
Social withdrawal	1		
Concealment of the illness	0.397^**^	1	
Perceived devaluation	0.525^**^	0.351^**^	1
SSI–F (PWUDs)	0.864^**^	0.649^**^	0.822^**^

** Correlation is significant at the 0.01 level (2–tailed).

## Discussion

This study was conducted to translate and evaluate the psychometric properties of SSI-F of PWUDs in Iran. In this study, at first, the cultural validation was carried out using the ten steps developed by Wilde et al. In this study, EFA with 140 subjects was used to examine the construct validity, and then the number of subjects is increased to 311, and CFA was carried out.

The results of EFA showed that the three factors account for about 57.114% of the variance of the 14 items, and 14 items and three factors were confirmed in effect. In the study conducted by Yildiz et al., the SSF-I with three factors and 14 items had been confirmed with a 66.6% variance of items in total (16). In this study, the first factor included 6 items, the second factor included 3 factors, and the third factor included 5 items where the results were the same as those of Yildiz et al. (16). In the study conducted by Yildiz et al. considering the structure of the questionnaire based on existing scales concerning the families of the patients suffering from mental disorders and focus group interview on 19 items, finally 5 items were removed considering the low factor loading (15). However, in this study, EFA was carried out on the 14-item scale developed by Yildiz et al., and it was carried out on families of PWUDs instead of families of individuals suffering from schizophrenia. Finally, the results of this study confirmed the same 3 factors with the 14 items in the SSF-I scale presented by Yildizet al. (16).

The results of CFA showed that the SSI-F of the PWUDs model with three factors in Iran has 14 items with an appropriate fit. In the study conducted by Yildiz et al., the SSI-F of the PWUDs model involved 3 factors and 14 items, and all fit indices were reported to be at the appropriate range (16). In explaining the results, it could be said that the above number of subjects was used in CFA, and the cultural similarities between Iran and Turkey can be one of the main factors resulting in the similarity in results.

The results of the study showed that the SSI-F of PWUDs of reliability coefficient was 0.891 and the stability of Cronbach's alpha was 0.879 in the range of 0.647 to 0.824. These results confirmed the acceptable reliability and stability of the test in the study population. The results of stability suggested by Yildiz et al. were consistent with the results of this study, in which Cronbach's alpha was found to be 0.88, and the reliability was reported to be 0.93 according to the test-retest method (16). This tool can be used for screening and evaluating the status of stigma in Iranian family members of PWUDs. Therefore, it can be used for teaching students and conducting research in the field of psychiatry, social sciences, and even family studies. In addition, it can be used in the rehabilitation process of PWUDs to support family members.

### Strengths and limitations of the study

Carrying out CFA and EFA on two separate populations was one of the strengths of this study. Unfortunately, we faced numerous problems due to the outbreak of coronavirus and the limitations help-seekers' families faced to participate in the study, and the sampling took more than 8 months. Moreover, due to the outbreak of coronavirus and the limitations of in-person contact with the subjects of the research, we resorted to having the questionnaires completed either in person in written form or electronically through WhatsApp and email.

## Conclusions

Generally, the results showed that the SSI-F of PWUDs in Iranian families was valid and reliable with three factors and 14 items, and it can be used to conduct relevant studies.

## Data availability statement

The original contributions presented in the study are included in the article/Supplementary materials, further inquiries can be directed to the corresponding author.

## Ethics statement

The studies involving human participants were reviewed and approved by Kermanshah University of Medical sciences Ethical Committe, the Ethical Code (IR.KUMS.REC.1399.999) was also received to conduct the study. The patients/participants provided their written informed consent to participate in this study.

## Author contributions

MD and AJ contributed to designing the study, AJ, MD, and AN collected the data, and data analyses were done by MD. The final report and article were written by AJ, MD, and AN. All authors participated and approved the study design. All authors read and approved the final manuscript.

## Funding

This study was drawn from a research project (No. 990955) sponsored by deputy of research and technology of KUMS. The cost of the payment is spent on the design and data collection of the study.

## Conflict of interest

The authors declare that the research was conducted in the absence of any commercial or financial relationships that could be construed as a potential conflict of interest.

## Publisher's note

All claims expressed in this article are solely those of the authors and do not necessarily represent those of their affiliated organizations, or those of the publisher, the editors and the reviewers. Any product that may be evaluated in this article, or claim that may be made by its manufacturer, is not guaranteed or endorsed by the publisher.

## Abbreviations

CVI: Content Validity Index
CVR: Content Validity Ratio
KMO: Kaiser Meyer Olkin
EFA: Exploratory factor analysis
CFA: Confirmatory Factor Analysis
TLI: Tucker-Lewis Index
NFI: Normed Fit Index
GFI: Goodness of Fit Index
RMSEA: Root Mean Square Error of Approximation
PC: Principal Components
SRMR: Standardized Root Mean Square Residual
SUD: Substance use disorder
PWUDs: Person Who Uses Drugs
SSI-F: Self-Stigma Inventory for Families
KUMS: Kermanshah University of Medical Sciences.
