# Corrigendum: Assessment of psychometric properties of the self-stigma inventory for Iranian families of persons who use drugs

**DOI:** 10.3389/fpubh.2023.1141748

**Published:** 2023-01-31

**Authors:** Mohammadreza Dinmohammadi, Amir Jalali, Arsalan Naderipour

**Affiliations:** ^1^Department of Nursing, School of Nursing and Midwifery, Zanjan University of Medical Sciences, Zanjan, Iran; ^2^Substance Abuse Prevention Research Center, Research Institute for Health, Kermanshah University of Medical Sciences, Kermanshah, Iran; ^3^Department of Emergency Medicine, Faculty of Paramedics, Kermanshah University of Medical Sciences, Kermanshah, Iran

**Keywords:** reliability, validity, stigma, drug user, family, substance use disorder

In the original article, there was an error in affiliation [3]. Instead of “Department of Emergency Medicine, Faculty of Paramedical, University of Medical Sciences, Kermanshah, Iran,” it should be “Department of Emergency Medicine, Faculty of Paramedics, Kermanshah University of Medical Sciences, Kermanshah, Iran.”

In the original article, Arsalan Naderipour's name was spelt incorrectly in the metadata. Instead of “Arsalan Naderipor” it should be, “Arsalan Naderipour.”

The authors apologize for this error and state that this does not change the scientific conclusions of the article in any way. The original article has been updated.

