# Supplementary material for: Assessment of psychometric properties of the self-stigma inventory for Iranian families of persons who use drugs
Source: Front Public Health. 2022 Nov 7;10:1017273. doi: 10.3389/fpubh.2022.1017273 (PMC9676927; doi:10.3389/fpubh.2022.1017273)
Supplement: Supplementary file 1 [file Table_1.DOCX]

**supplementary Table 1. Rotated Component Matrix^a^**

|  | Component | | |
| --- | --- | --- | --- |
|  | 1 | 2 | 3 |
| Q1 | .122 | .725 | .212 |
| Q2 | .387 | .438 | .339 |
| Q3 | .450 | .567 | .206 |
| Q4 | .447 | .470 | -.046 |
| Q5 | .312 | .728 | .184 |
| Q6 | .143 | .767 | .060 |
| Q7 | .242 | .148 | .674 |
| Q8 | .218 | .048 | .813 |
| Q9 | -.015 | .170 | .686 |
| Q10 | .627 | .264 | .279 |
| Q11 | .642 | .232 | .282 |
| Q12 | .723 | .339 | .015 |
| Q13 | .809 | .131 | .199 |
| Q14 | .699 | .131 | .071 |
| Extraction Method: Principal Component Analysis.  Rotation Method: Varimax with Kaiser Normalization. | | | |
| a. Rotation converged in 5 iterations. | | | |
